# Cell Line Secretome and Tumor Tissue Proteome Markers for Early Detection of Colorectal Cancer: A Systematic Review

**DOI:** 10.3390/cancers9110156

**Published:** 2017-11-16

**Authors:** Megha Bhardwaj, Vanessa Erben, Petra Schrotz-King, Hermann Brenner

**Affiliations:** 1 Division of Preventive Oncology, German Cancer Research Center (DKFZ) and National Center for Tumor Diseases (NCT), 69120 Heidelberg, Germany; megha.bhardwaj@nct-heidelberg.de (M.B.); vanessa.erben@nct-heidelberg.de (V.E.); petra.schrotz-king@nct-heidelberg.de (P.S.-K.); 2 Division of Clinical Epidemiology and Aging Research, German Cancer Research Center (DKFZ), 69120 Heidelberg, Germany; 3 German Cancer Consortium (DKTK), German Cancer Research Center (DKFZ), 69120 Heidelberg, Germany

**Keywords:** secretome, tumor tissue proteome, colorectal cancer, sensitivity, specificity, early diagnosis

## Abstract

*Objective:* In order to find low abundant proteins secretome and tumor tissue proteome data have been explored in the last few years for the diagnosis of colorectal cancer (CRC). In this review we aim to summarize the results of studies evaluating markers derived from the secretome and tumor proteome for blood based detection of colorectal cancer. *Methods:* Observing the preferred reporting items for systematic reviews and meta-analysis (PRISMA) guidelines PubMed and Web of Science databases were searched systematically for relevant studies published up to 18 July 2017. After screening for predefined eligibility criteria a total of 47 studies were identified. Information on diagnostic performance indicators, methodological procedures and validation was extracted. Functions of proteins were identified from the UniProt database and the the Quality Assessment of Diagnostic Accuracy Studies-2 (QUADAS-2) tool was used to assess study quality. *Results:* Forty seven studies meeting inclusion criteria were identified. Overall, 83 different proteins were identified, with carcinoembryonic Antigen (CEA) being by far the most commonly reported (reported in 24 studies). Evaluation of the markers or marker combinations in blood samples from CRC cases and controls yielded apparently very promising diagnostic performances, with area under the curve >0.9 in several cases, but lack of internal or external validation, overoptimism due to overfitting and spectrum bias due to evaluation in clinical setting rather than screening settings are major concerns. *Conclusions:* Secretome and tumor proteome-based biomarkers when validated in blood yield promising candidates. However, for discovered protein markers to be clinically applicable as screening tool they have to be specific for early stages and need to be validated externally in larger studies with participants recruited in true screening setting.

## 1. Introduction

With approximately 1.4 million incident cases per year and 700,000 deaths, colorectal cancer (CRC) is the third most common malignancy and fourth leading cause of cancer-related mortality worldwide [1]. Additionally, CRC is one of major contributors to disability-adjusted-life-years (DALYs) in most regions of the world [2]. Recent estimates suggest that the burden of CRC is expected to increase by 60%, to more than 2.2 million new cases and 1.1 million deaths by 2030 [3]. A large proportion of this burden could be prevented by screening, either by detection of the cancer at earlier stages, when chances of cure are substantially higher than at later stages or by detection and removal of precancerous lesions, e.g., through screening by flexible sigmoidoscopy or colonoscopy [4,5].

Nevertheless, the use of endoscopic exams for primary screening faces limitations in terms of invasiveness, available capacities, costs, inconvenience and adherence [6,7]. As a non-invasive alternative, stool-based tests, in particular fecal immunochemical tests for hemoglobin (FITs) are increasingly used, but studies consistently show that people would highly prefer blood-based screening test over stool-based screening tests [8,9,10,11]. The already known blood-based protein biomarkers like carcinoembryonic antigen (CEA) and carbohydrate antigen 19-9 (CA 19-9) alone lack the sensitivity and specificity needed for early diagnosis of CRC [12,13,14] which underlines the importance to identify and validate additional, more sensitive blood-based markers or marker signatures.

In recent years the secretome from conditioned media of cell culture and the tumor tissue proteome have turned out to be interesting and informative sources for markers associated with early diagnosis of CRC. The cell line secretome and tumor tissue proteome comprise all proteins shed, secreted or leaked from the cancer cells [15,16]. These secreted or leaked proteins are likely to end up in the systemic blood circulation and even though concentrations may be low, they have a high potential for serving as candidate diagnostic markers. The aim of this systematic review is to provide an overview of studies that investigated the diagnostic potential of proteins from the cell line sercretome and/or tumor tissue and validated those markers in blood from CRC patients and controls.

## 2. Methods

### 2.1. Data Sources and Search Strategy

Adhering to preferred reporting items for systematic reviews and meta-analysis (PRISMA) guidelines [17] a literature search was performed in the online scientific citation databases Web of Science (previously known as ISI Web of Knowledge) and MEDLINE (via PubMed) from establishment until 18 July 2017. Details of the search terms which included keywords like “Cell line”, “Secretome”, “Tumor microenvironment”, “Tumor tissue”, “Protein”, or “Colorectal Cancer” are reported in the Supplementary Materials File S1.

### 2.2. Study Selection, Data Extraction and Risk of Bias Assessment

The language of the selected literature was restricted to English. Only studies which firstly discovered protein markers in the cell-line secretome or tumor tissue proteome and later validated these candidates in serum or plasma were included. Studies were excluded if they did not report sufficient information on diagnostic indicators, if the candidates were not validated in blood, if the number of CRC cases were less than 10 and if the diagnostic performance was reported only for unidentified peaks. A set of proteins was classified as combination if the joint diagnostic performance was reported for ≥2 proteins in an individual study.

Data from all relevant studies regarding characteristics of study participants, platform used for quantification in blood, diagnostic performance related indicators and methods used for validation and correction for overoptimism was extracted from the main and Supplementary Files by Megha Bhardwaj and Vanessa Erben (two authors) independently. In order to assess the risk of bias the Quality Assessment of Diagnostic Accuracy Studies 2 instrument (QUADAS-2) [18] was used. The information on functions and locations of proteins was extracted from the UniProt database [19] and 95% confidence intervals (95% CI) of given sensitivities and specificities by Clopper-Pearson method were calculated using R (R core team 2016, version R 3.3.2, Vienna, Austria).

## 3. Results

### 3.1. Study Selection

**Figure 1:**
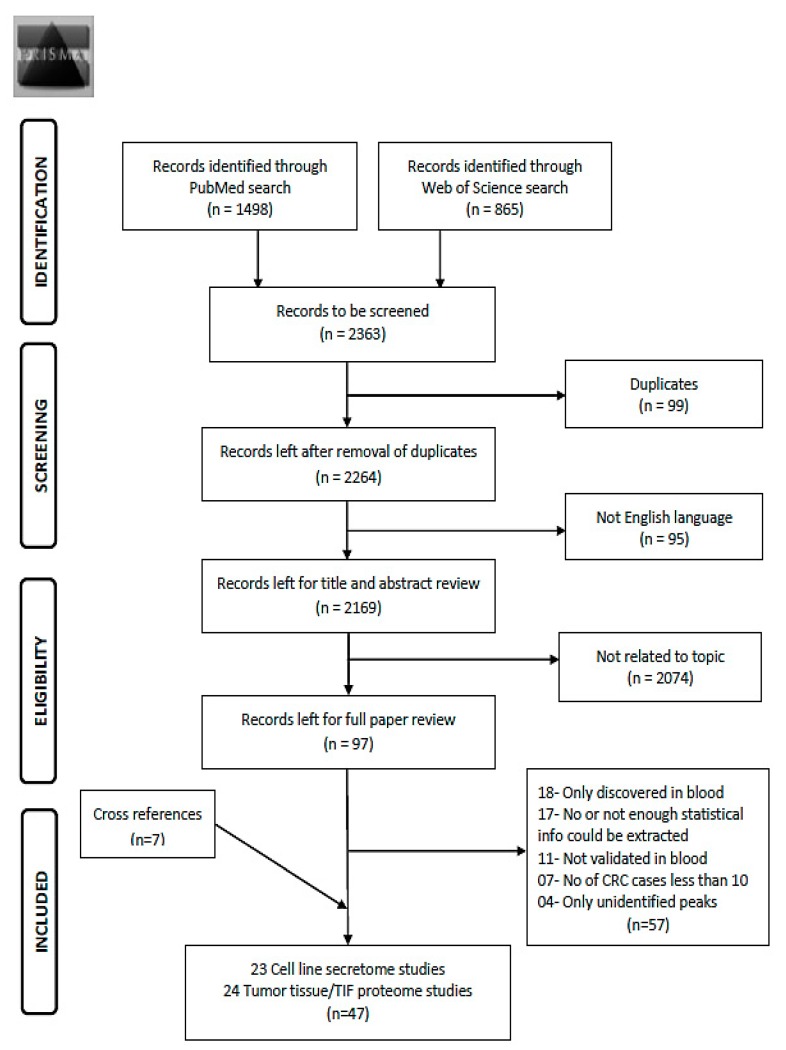
Preferred reporting items for systematic reviews and meta-analysis (PRISMA) Flow diagram for literature search process for records identified via PubMed and Web of Science database [20]. Abbreviations: TIF: tissue interstitial fluid; CRC: colorectal cancer.

**Figure 2:**
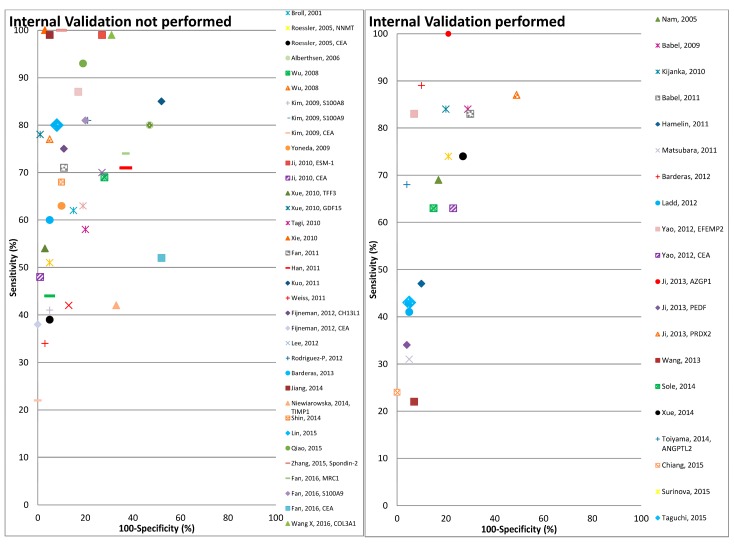
Graphical representation of diagnostic performance of protein markers with respect to internal validation performed by the studies.

**Figure 3:**
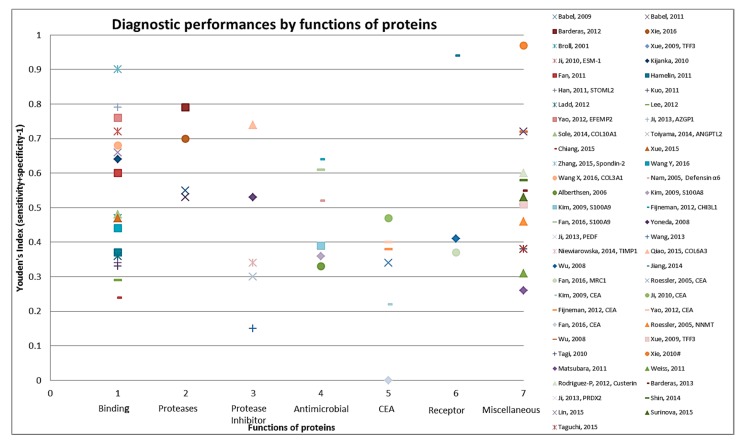
Graphical representation of diagnostic performance of protein markers with respect to molecular function of these protein markers.

Using the search term given in Supplementary Materials File S1, 2363 records were obtained from the two databases (Figure 1). After exclusion of duplicates (*n* = 99) and non-English articles (*n* = 95), the remaining 2169 articles were reviewed for title and abstract. From these, 2074 articles were found to be not related to the topic and 97 articles remained for full text review. Applying the pre-determined eligibility criteria, 40 studies remained and seven other studies were acquired with careful cross referencing. Amongst the 47 finally selected studies, 29 reported diagnostic performance of combinations of protein markers and 18 studies reported diagnostic performance of individual markers. Twenty-three studies assessed cell line secretome, seventeen out of which additionally assessed tumor tissues. The remaining 21 studies were exclusively based on tumor and normal tissue comparison and three studies on xenograft tissue interstitial fluid (TIF). Characteristics of the study populations for cell line secretome and tumor tissue/TIF studies are reported in Table 1 and Table 2 respectively. Furthermore, information concerning type of proteins, platform for validation in blood, internal and/or external validation employed and diagnostic performance related indicators are summarized in Table 3 and Table 4 for cell line secretome and tumor tissue/TIF studies respectively. Figure 2 and Figure 3 represent the diagnostic performances of proteins by internal validation performed and molecular functions, respectively. Supplementary Materials S1–S5 contain search strategy, stage specific numbers and diagnostic performances, functions and locations of proteins, risk of bias and applicability concerns for individual studies and the PRISMA checklist [20], respectively.

### 3.2. Study Characteristics

Table 1 and Table 2 present an overview on the characteristics of the participants for the cell line secretome and tumor tissue proteome marker studies, respectively. Twenty-nine studies were conducted in Asia; fourteen in China [21,22,23,24,25,26,27,28,29,30,31,32,33,34], five each in Taiwan [35,36,37,38,39] and Japan [40,41,42,43,44], four in Korea [45,46,47,48] and one in Singapore [49]. Out of 15 studies that were carried out in Europe, six studies were performed in Spain [50,51,52,53,54,55], four in Germany [56,57,58,59] out of which one multicenter study was also carried out in the Czech Republic [58], and one each in Denmark [60], France [61], Ireland [62], The Netherlands [63] and Poland [64]. Three studies were carried out in the United States of America [65,66,67]. Among the 23 different cell secretome studies cell line SW480 was used in 16, SW620 in 12, HCT116 in 11, Colo205 in 10 and Caco-2, LoVo and HT-29 each in eight different studies, respectively. For 24 studies that used tumor tissue and pairwise or adjacent normal mucosa or TIF from xenograft models, the number of tumor tissue samples ranged from 6 to 294.

The numbers of study participants used for validating the candidates in blood samples ranged from 30 to 280 CRC cases and from 20 to 201 controls for marker combination studies and from 8 to 405 CRC cases and 16 to 317 controls for individual protein marker studies. In only one study blood was collected from asymptomatic participants before diagnosis [65]. In one study blood was collected post-operatively [40]. All studies had a case control design and nine studies matched cases and controls with respect to age [21,32,39,43,48,52,54,60,65] and/or gender [32,43,48,52,60,65]. In some of the studies, mean age varied strongly between cases and controls e.g., 39 versus 67 years in a study from Japan [40] and 65.5 versus 41 years in a study from China [24]. There were more male than female study participants in 30 out of 34 studies where the gender distribution was reported. Stage distribution of cases was reported by 36 different studies and there was a majority of early stage cases (i.e., stage I or II) in 17 studies and late stage cases (i.e., stage III or IV) in 16 studies whereas early and late stages were equally represented in three studies. Nine studies used other case groups like participants with adenomas (details in Supplementary Materials Table S2).

### 3.3. Diagnostic Performance of Biomarkers

Overall the diagnostic performance of all markers from the 47 studies included in the review varied widely, with the sensitivity, specificity and Area under the ROC curve (AUC) ranging from 22–100%, 48–100% and 0.464–0.989 respectively (Table 3 and Table 4). The number of proteins evaluated ranged from 1 to 12. Eighteen out of 47 studies performed some form of correction for overoptimism. In Figure 2 the diagnostic performances of the studies are plotted (sensitivity on *y*-axis and 1-specificty on *x*-axis) with respect to internal validation performed by these studies for correction of overoptimism. As shown in this figure the studies with internal validation typically reported somewhat lower sensitivities at comparable levels of specificity, than studies without internal validation.

As shown in Table 3, fifteen studies that used cell line secretome for identification of combination protein markers, reported 31 different protein marker candidates. The number of protein markers in the combinations varied from 2 to 12. The most common marker included in the combinations by far was CEA (included in 10 out of 15 combinations). Out of the 15 studies only six [37,50,51,52,65,67] performed some form of correction for overoptimism. From these six the best diagnostic performance with 89% sensitivity at 90% specificity, was reported by Barderas et al. [52] that used secretome from 16 different cell lines and Enzyme-linked immunosorbent assay (ELISA) based validation for a combination of protein markers MAPKAPK3, ACVR2B, PIM1, FGFR4 and phages GRN, NHSL1, SREBF2.

In total 14 of the studies comparing tumor tissues and adjacent normal mucosa reported some biomarker combinations (Table 4). Overall, 38 different proteins were included in some of the combinations, with CEA included in eight out of 14 combinations, again being the most commonly reported. From six studies that performed some form of correction for overoptimism [25,29,42,58,61,62], Kijanka et al. [62] reported best diagnostic performance (84% sensitivity at 80% specificity) with a 12 protein marker combination using tissue from 43 CRC patients and 19 controls with no neoplasm of colon.

From the 18 studies that reported individual biomarkers rather than combinations, eight used cell line secretome and 10 used tumor tissue proteome data. Twenty-three different individual biomarkers were reported by this category and except CEA (reported in six of the 18 studies) no biomarker was used in more than one study. Only five studies [22,30,43,55,66] performed correction for overoptimism, all utilizing the split sample method and among these the best diagnostic performance was reported by Ji et al. [22] for protein Zinc-α-2-glycoprotein with 100% sensitivity at 79% specificity.

### 3.4. Functionality of Discovered Proteins

The 83 identified protein biomarker candidates identified from the cell line secretome and tumor tissue proteome represent different classes of proteins. Apart from CEA (identified in 24 studies), only few proteins have been identified in more than one study (CEA in 24 studies, CA 19-9 in four studies, S100A9 and LRG1 in three studies each and ACVR2B, AZGP1, GDF15, MAPKAPK3, PIM1 in two studies each). Supplementary Materials Table S3 provides information on the molecular and biological function and location with cellular component of the proteins as identified from the UniProt database [19]. Thirty-nine proteins function as binding (DNA-, RNA-, metal-ion- or ATP-) proteins, while eight are protease inhibitors, followed by seven each that function as antimicrobial. Overall, a large variety of biological functions were represented. For example, eleven proteins were found to be involved in apoptosis and in cell adhesion each, eight in inflammatory response and other six proteins in transcription. As shown in Figure 3 when diagnostic performances of proteins were represented by molecular function of these markers the best performances were seen for some of the binding (DNA-, RNA-, metal-ion- or ATP-) proteins and proteases, followed by protease inhibitors and antimicrobials.

Various and sometimes multiple methods were used for identification of proteins in cell line secretome of tumor tissue proteome (Table 1 and Table 2) and for validation of those markers in blood (Table 3 and Table 4). Immunohistochemistry (IHC) was used individually or in combination with other methods for identification of proteins in the cell line secretome or tumor tissue proteome in 24 different studies. Fifteen studies used a Mass spectrometry based platform and nine studies used various types of microarray (antibody/protein/phage/tissue/reverse phase) based platforms. For 39 out of 47 studies ELISA was used to validate biomarkers in blood.

### 3.5. Assessment of Risk of Bias across Biomarker Combination Studies

Results of our assessment for risk of bias and applicability using the QUADAS-2 tool are summarized in Supplementary Materials Table S4. In the QUADAS-2 assessment for four domains, no single study out of the 47 studies presented low risk of bias and low applicability concerns in all domains. Risk of bias was highest for the domain “Patient selection” with “High” risk for all of the studies except one [65]. This is because the blood sample from CRC cases was not collected in a true screening setting from asymptomatic participants in these studies. Risk of bias for “Index test” was “High” for more than half of the studies (*N* = 25) because a pre-specified cut-off was not used by these studies and no correction for overoptimism was performed. Risk of bias for Reference standard was also “High” for 26 studies since the reference standard was not clearly set for both CRC cases and controls in these studies. For nearly all studies it is unclear whether the results of the index test were interpreted without knowledge of results of the reference standard and if the samples were blinded before the execution of the index test or not. For none of the studies the time from storage to analysis was specified.

## 4. Discussion

In this review we present a comprehensive overview of studies that searched cell line secretome or tumor tissue proteome markers for early detection of colorectal cancer. We focused only on studies that validated the protein markers in blood. We summarize results of 47 studies which investigated 83 proteins. Overall sample size varied from 42 to 427 and was less than 100 in 18 studies, between 100 and 200 in 13 studies and more than 200 in 16 studies. The age of CRC patients ranged from 19 to 93 years and 17 to 93 years for controls. The majority of participants were male in 30 studies. Even though 36 out of 47 studies specified the stage wise distribution of cases, only thirteen out these specified stage wise diagnostic performance of the protein markers. Diagnostic performances reported for these markers were highly diverse ranging from sensitivity (at specificity) 22% (93%) to 100% (97%). In only one study [65] validation of the protein markers was performed in a true screening setting, where they showed rather limited diagnostic performance (41% sensitivity at 95% specificity and AUC = 0.724).

Twenty two most abundant proteins dominate 99% of total protein mass in plasma and the vast dynamic concentration range of these proteins makes quantitation of low abundant proteins difficult in blood [68]. There are around ten orders of magnitude in concentration between most abundant proteins in blood and tumor cell proteins [69]. However comparative analysis between tumor tissue proteome and adjacent normal mucosa can reveal potential candidates for diagnosis because certain secretary proteins are produced by various cell types of the same tissue [70]. As not all proteins altered in tumors are secreted in same concentrations in blood, identifying candidate proteins in the tumor tissue proteome or cell line secretome and subsequently validating them in blood with high sensitivity assays appears to be a rational and promising approach. Moreover tumors comprise of numerous neoplastic and non-neoplastic cells but isolating particular cells for candidate identification from the tumor tissue can be a daunting task especially due to often small size of biopsied tissue. In the current review several candidates were identified from this type of comparison and yielded diagnostic performances, which, if confirmed in yet to be conducted validation studies in true screening settings, might result into clinically applicable tests.

Commonly used substitutes for neoplastic tumor tissue are the immediately available numerous CRC cell lines representing various stages and histotypes of tumor. Cell lines can be easily manipulated and are well suited for isolation of secretome by fractionation which helps reduce the sample complexity and aids identification not only of abundant proteins but also proteins present in intermediate and low quantities. Secretome studies can minimize the biological, environmental or behavioral variability which makes results quantifiable and reproducible [71,72]. However, there are several technical difficulties associated with secretome analysis like its contamination by cell media or intra-cellular proteins and also the high dilution factor of proteins in cell culture media [71]. Nevertheless, in current review studies that used cell lines reported several candidates with apparently very promising diagnostic performance in case-control studies conducted in clinical settings. Again validation and potential confirmation in asymptomatic screening participants will be crucial to determine their potential use as screening markers.

A major concern in biomarker studies, especially in studies on signatures of multiple biomarkers in overfitting and the resulting overoptimism of derived measures of diagnostic performance. Application of internal and/or external validation is crucial to overcome such overestimation of diagnostic performance. In the current review 18 studies performed some form of correction for overoptimism out of which 11 used the split sample method, four and three studies used bootstrap and cross validation methods, respectively. However, external validation of these candidates on participants differing from setting, origin and time frame than the original population was not performed by any study. Since none of the markers were externally validated, validating seemingly promising candidate markers or marker algorithms in different study populations are warranted. In particular, validation in true screening settings, where the majority of cases are typically of early stages is indispensible for deriving valid estimates of expected diagnostic performance for screening purposes. Such validation studies should also take utmost care to avoid other potential common biases, such as clinical review and detection bias [73,74] for which blinding samples is most crucial, or it might result in bias resulting from potentially different handling, storage or preprocessing of blood samples from cases and controls should also be accounted for.

To our knowledge, this is the first systematic review summarizing the existing literature on CRC secretome and tumor proteome biomarkers validated in blood. In addition to diagnostic performances, particular attention was given to epidemiological aspects, precautions against possible biases and functional aspects of identified biomarkers. A number of limitations of our review have to be considered like despite comprehensive search in two well established databases and comprehensive cross referencing, we cannot exclude the possibility of having missed potentially relevant studies in particular if they were not reported in English or reported in the grey literature. Additionally, the differences in the functionality type and numbers of proteins, diversities in types of study populations and experimental techniques, differences in cell lines and in number of tumor tissue and in the blood collection or time to storage procedures or time to analysis and varied statistical evaluation procedures makes it impossible to make a direct balanced comparison or conduct meta-analysis of all the individual marker or marker signatures from 47 different studies. Also, correlations between identified secreted markers and mutation status of tumors cannot be established from the reviewed studies.

Despite its limitations, our systematic review illustrates the large potential of cell line secretome and tumor tissue derived protein biomarkers, in particular biomarker signatures consisting of multiple proteins, might have for blood based detection of CRC. Our review also demonstrates, however, that for most of the identified markers and signatures, in particular those with the apparently most promising diagnostic performance, rigorous validation in true screening settings is still required, paying particular attention to standardized pre-diagnostic collection, blinded processing and analysis among CRC cases and controls, which should be comparable in all aspects except the presence of CRC, as well as rigorous control for overoptimism by internal and/or external validation. Ideal settings for this purpose are studies among participants of screening colonoscopy, with blood samples taken prior to colonoscopy. Validation studies should also be sufficiently large in order to provide accuracy estimates with reasonable precision which is often a major challenge for screening colonoscopy studies due to the typically low prevalence of CRC cases in this setting. Our systematic review may help to select markers to be included in such validations, either individually or in combination (with combinations not necessarily restricted to the previously evaluated ones). If some of the apparently very promising diagnostic performance results for these markers can be successfully validated and confirmed in such settings they might become promising alternatives for non-invasive CRC screening. Additional factors to be considered in this context, in particular when compared with other screening options and preferably in the context of comprehensive modeling of screening effectiveness and cost-effectiveness, will be adherence to and costs of blood based tests.

## Figures and Tables

**Table 1:** Study characteristics of the cell line secretome studies.

First Author, Year, Country [Ref]	Platform	Cell Lines Used											Study Sample (Tissue)	Study Samples (Blood)			
		SW480	SW620	HCT-116	Colo205	Caco-2	HT-29	LoVo	SW48	HCT-15	Colo320	Others		Sample Size(N) CRC Cn	% Late Stage (III/IV) CRC	Mean Age (Range/±SD) CRC Cn	% Males CRC Cn
Combination Marker Studies																	
Wu, 2008, Taiwan [35]	MALDI, SDS	x			x							-	169 paired CRC & NC	201 201	50	63.5 (24–88) (24–84)	52 55
Wu, 2008, Taiwan [36]	MALDI, IHC	x	x		x							1	241 CRC & 231 NM	280 147	60	60.1 (19–88) (24–84)	50 55
Babel, 2009, Spain [50]	PrMA, IHC	x		x	x				x	x		3	45 paired CRC & NM	52 42	62	71 (±10.6) 61.7 (±16.0)	62 60
Tagi, 2010, Japan [40]	qRT-PCR, IHC	x		x	x	x	x	x		x	x	7	130 CRC	105 100	44	39.2 (23–59) 66.8 (26–93)	-
Babel, 2011, Spain [51]	PhMA, IHC	x		x	x	x	x		x	x	x	8	25 paired CRC & NM	50 46	66	70.8 (41–91) 59.6 (34–89)	66 63
Fan, 2011, Taiwan [38]	qRT-PCR, WB, IHC	x	x		x							-	10 CRC &NC/64 CRC	86 72	59	-	-
Weiss, 2011, Germany [59]	SDS, IHC		x									4	4 paired CRC & NM	59 45	75	(37–87) (17–87)	75 49
Barderas, 2012, Spain [52]	PhMA, WB	x		x	x	x	x		x	x	x	8	6 paired CRC & NM	50 46	66	70.8 (±15.7) 60.9 (±11.4)	66 61
Ladd, 2012, USA [65]	LC/MS-MS	x			x							-	-	32 32	47	68.1 67.8	0 0
Lee, 2012, Korea [48]	IHC, WB	x	x	x			x	x				6	120 paired CRC & NM	219 75	47	--	63 63
Barderas, 2013, Spain [53]	MS, SDS											2	-	40 20	68	67.5 (43–85) 60.5 (49–88)	58 60
Shin, 2014, Korea [46]	LC/MS-MS			x								1	-	228 77	40	62.6 (±8.7) 68.2 (±7.0)	60 43
Chiang, 2015, Taiwan [37]	qRT-PCR, IHC	x	x		x							-	132 paired CRC & NC	120 120	49	65.3 (±12.7) 45.3 (±10.1)	61 51
Lin, 2015, Singapore [49]	MS, WB			x								-	-	45 47	67	- -	33 -
Taguchi, 2015, USA [67]	AbMA, MS	x	x	x	x	x	x	x	x			-	66 CRC & 20 NM	60 60	50	- 55.7 (±9.9)	40 73
Individual Marker Studies																	
Nam, 2004, USA [66]	MA, WB	x	x	x				x				2	36 paired CRC & NM	49 18	-	-	-
Xue, 2010, China [28]	LC/MS-MS	x	x									-	69 CRC	144 156	53	59 (27–88) 56 (25–81)	53 54
Rodriguez-Pineiro, 2012, Spain [54]	MALDI					x						-	-	31 33	39	69.0 (± 9.0)	-
Toiyama, 2014, Japan [43]	qRT-PCR, IHC	x				x	x	x				1	195 paired CRC & NM	195 45	47	66.7 (±10.7) 57.2 (±13.4)	58 56
Qiao, 2015, China [24]	LC-MS, WB		x				x	x				5	90 paired CRC & NM	42 48	-	65.5 (±10.97) 41 (±16.28)	67 44
Zhang, 2015, China [33]	qRT-PCR, WB, IHC		x	x		x		x			x	-	90 paired CRC & NC	43 38	-	-	-
Fan, 2016, China [31]	LC/MS-MS	x	x				x					3	-	112 96	37	58.9 (±7.94) 61.5 (±7.94)	
Wang X, 2016, China [26]	MA, WB, IHC	x	x	x		x		x				-	90 CRC & matched NM	86 68	-	-	-

Note: Age is in years; -: means not specified by the study; x: this type of cell line used by the study. Abbreviations: CRC: Colorectal Cancer; Cn: Controls; NC: Normal colonic tissue; NM: Normal mucosa. AbMA: Antibody microarray; IHC: Immunohistochemistry; LC-MS: Liquid chromatography-mass spectrometry; LC/MS-MS: Liquid chromatography-tandem mass spectrometry; MA: Microarray; MALDI: Matrix-assisted laser desorption/ionization; MS: Mass spectrometry; PhMA: Phage microarray; PrA: Protein array; PrMA: Protein microarray; qRT-PCR: Quantitative reverse transcription polymerase chain reaction; SDS: SDS PAGE or sodium dodecyl sulphate-polyacrylamide gel electrophoresis; SPM: Spectrophotometrically with protein assay; WB: Western Blot.

**Table 2:** Patient characteristics of the tumor tissue proteome/TIF studies.

First Author, Year, Country, [Ref]	Platform	Study Samples (Tumor Tissue)				Study Samples (Blood)				
		Sample Size (N) Cases NM	% Late Stage (III/IV) CRC	Mean Age (Range/±SD) CRC	% Males CRC	Sample Size (N) CRC Cn	% Late Stage (III/IV) CRC	Mean Age (Range/±SD) CRC Cn		% Males CRC Cn
Combination Marker Studies										
Broll, 2001, Germany [56]	SPM	38 paired CRC & NC	-	-	-	122 65	44	68.3 (32–92) 66.4 (27–89)		49 48
Alberthsen, 2006, Denmark [60]	SELDI-TOF MS	32 CRC tumors	50	-	63	119 34	49	- (50–80)		56 53
Yoneda, 2009, Japan [41]	MA, IHC	-	-	-	-	159 40	42	(38–90) (29–85)		67 53
Kijanka, 2010, Ireland [62]	IHC	43 CRC 19 NM	-	-	-	43 40	-	-		-
Xie, 2010, China [27]	GeMS, TiMA	93 paired CRC & NC	3	66.05 ± 13.89	53	42 42	-	-		-
Hamelin, 2011, France [61]	MALDI, IHC	20 paired CRC & NC	70	-	50	112 90	50	70 (± 11) 58 (± 4)		54 60
Kuo, 2011, Taiwan [39]	IHC	104 paired CRC & NM	92	-	47	59 52	58	-		58 -
Matsubara, 2011, Japan [42]	IHC, RPPM	20 tissues	-	-	-	26 87	35	63.0 (±12.0) 43.0 (±16.0)		50 64
Wang, 2013, China [25]	MALDI, IHC	248 CRC 75 NM	-	-	-	143 85	46	54.7 (±1.02) 52.5 (±1.19)		58 51
Jiang, 2014, China [23]	IHC	98 CRC 30 NC	61	(28–88)	62	182 101	-	61 (23–86) (21–62)		52 45
Surinova, 2015, Germany, CR [58]	LC/MS-MS	16 paired CRC & NC	25	62.3 ± 9.5	81	202 67	50	67 (59–74.8) 49 (52–65)		56 76
Xue, 2014, China [29]	WB	190 paired CC & NC	45	66 (22–95)	43	120 40	37	66.8 (26–93) 39.2 (23–59)		41 -
Wang Y, 2016, China [32]	LC/MS-MS	TIF 16 AOM-DSS mice	-	-	-	16 16	56	(35–67)		56
Xie, 2016, China[34]	LC/MS-MS	TIF Apc^Min/+^ & WT mice	-	-	-	30 30	-	(31–70) (31–70)		60 57
Individual Marker Studies										
Roessler, 2005, Germany [57]	SDS	18 paired CRC & NM	28	73.3 (±9.7)	61	109 317	40	- (12–89)	-	
Kim, 2009, Korea [47]	2-D DIGE, MS, IHC	6 paired CRC & NM	33	53.5 (±11.4)	50	77 21	52		-	
Ji, 2010, Korea [45]	MA, WB, IHC	66 paired CRC & NM	-	-	-	100 78	49	-	61 -	
Han, 2011, China [21]	LC/MS-MS	28 paired CRC & NM	61	62.2 (±14.3)	57	70 70	-	-	-	
Fijneman, 2012, The Netherlands [63]	GeLC/MS-MS	TIFFabpl*Cre;apc*^15lox/+^C57Bl/6 mice	-	-	-	8 36	63	71.8 (±6.4) 60.2 (±13.8)	50 42	
Hosono, 2012, Japan [44]	IHC	62 adenoma	-	-	-	62 ^A^ 34	-	67.7 (±8.2) 67.6 (±15.9)	63 59	
Yao, 2012, China [30]	IHC	88 CRC 16 NM	41	(35–88)	57	122 79	-	-	- -	
Ji, 2013, China [22]	IHC, LC/MS-MS	294 CRC	71	59.55 ± 12.34	55	405 84	48	-	55 56	
Niewiarowska, 2014, Poland [64]	IHC	38 CRC tissues	-	-	-	43 24	88	67.1 (±1.89) 55.7 (±7.3)	63	
Sole, 2014, Spain [55]	LC/MS-MS	70 CRC 34 NM	0	62.5 (50–69)	23	80 77	55	67 (34–89) 66 (22–83)	65 51	

Note: Age is in years; -: means not specified by the study; ^A^: The study population consisted of Adenoma cases only. Abbreviations: CRC: Colorectal Cancer; Cn: Controls; 2-D DIGE: 2D-differential gel electrophoresis; GeLC/MS-MS: Nano-liquid chromatography-tandem mass spectrometry; GeMS: In-gel digestion coupled with mass spectrometric analysis; IHC: Immunohistochemistry; LC/MS-MS: Liquid chromatography-tandem mass spectrometry; MA: Microarray; MALDI: Matrix-assisted laser desorption/ionization; NC: Normal colonic tissue; NN: Nonneoplastic; NM: Normal mucosa; RPPM: Reverse phase protein microarray; SDS: SDS PAGE or sodium dodecyl sulphate-polyacrylamide gel electrophoresis; SELDI-TOF MS: surface-enhanced laser desorption ionization time of flight mass spectrometry; SPM: Spectrophotometrically with protein assay; TiMA: Tissue microarray; TIF: Tissue interstitial fluid; WB: Western Blot.

**Table 3:** Diagnostic performance of cell line secretome markers validated in blood.

First Author, Year, [Ref]	Proteins											Sensitivity (95%CI) %	Specificity (95%CI) %	Area under the Curve (95%CI)	Method	Validation
	CEA	CA19-9	ACVR2B	AZGP1	GDF15	LRG1	S100A9	MAPKAP3	TIMP1	PIM1	Others					
Cell Line Secretome Combination Marker Studies																
Wu, 2008 [35]	x										CRMP2	77 (71–83)	95 (91–98)	0.75 (0.68–0.78)	ELISA	No
Wu, 2008 [36]	x										Mac-2BP	69 (63–74)	72 (64–79)	0.774 (0.727–0.816)	ELISA	No
Babel, 2009 [50]			x					x		x		84 (71–93)	71 (55–84)	0.85	ELISA	BS
Tagi, 2010 [40]		x									DK, S-p53 (Stg.1)	58 (48–68)	80 (71–87)	-	ELISA	No
Babel, 2011 [51]											SULF1, MST1/STK4 & phages NHSL1, GTF2i, SREBF2, GRN	83 (70–92)	70 (55–83)	0.86	ELISA	BS
Fan, 2011 [38]	x										SEC61β	71 (60–80)	89 (79–95)	0.838 (0.774–0.903)	ELISA, WB	No
Weiss, 2011 [59]	x										sE-cadherin	34 (22–48)	97 (87–100)	-	ELISA	No
Barderas, 2012 [52]			x					x		x	FGFR4 & phages GRN, NHSL1, SREBF2	89 (77–96)	90 (78–97)	0.925	ELISA	BS
Ladd, 2012 [65]	x					x					IGFBP2, MAPRE1	41 (24–60)	95 (81–100)	0.724	ELISA	SS
Lee, 2012 [48]	x										HMGB1	42 (35–49)	87 (77–94)	0.643	ELISA	No
Barderas, 2013 [53]					x		x				S100A8/A9, SERPINI1	60 (43–75)	95 (75–100)	0.884	ELISA	No
Shin, 2014 [46]	x										PAI-1, TRFM	68 (62–74)	90 (81–96)	0.821 (0.731–0.890)	ELISA	No
Chiang, 2015 [37]	x										BST2	24 (17–33)	100 (97–100)	0.872 (0.828–0.916)	ELISA	SS
Lin, 2015 [49]	x										LAMB1	80 (65–90)	92 (80–98)	0.911 (0.85–0.97)	ELISA	No
Taguchi, 2015 [67]	x										MAPRE, AK1	43 (30–56)	95 (86–99)	-	ELISA	BS
Cell Line Secretome Individual Marker Studies																
Nam, 2005 [66]											Defensin α6	69 (54–81)	83 (58–96)	-	ELISA	SS
Xue, 2010 [28]					x						TFF3	54 (46–62) 78 (70–85)	97 (93–99) 99 (96–100)	0.730 (0.670–0.791) 0.897 (0.856–0.938)	ELISA	No
Rodriguez-P, 2012 [54]											Clusterin	81 (63–93)	79 (61–91)	0.845 (0.747–0.942)	ELISA	No
Toiyama, 2014 [43]											ANGPTL2	70 (63–76)	96 (86–100)	0.885 (0.838–0.923)	ELISA	SS
Qiao, 2015 [24]											COL6A3	93 (81–99)	81 (67–91)	0.885	ELISA	No
Zhang, 2015 [33]											Spondin-2	100 (92–100)	90 (76–97)	0.959	ELISA	No
Fan, 2016 [31]	x						x				MRC1	74 (65–82) 81 (73–88) 52 (42–62)	63 (53–73) 80 (71–88) 48 (38–58)	0.744 (0.678–0.810) 0.873 (0.826–0.920) 0.464 (0.384–0.544)	ELISA	No
Wang X, 2016 [26]	x										COL3A1	99 (94–100) 70 (59–79)	69 (57–80) 73 (61–83)	0.92 0.791	ELISA	No

Abbreviations: BS: Bootstrap method; ELISA: Enzyme-linked immunosorbent assay; SS: Split Sampling method; WB: Western Blot. Abbreviations for proteins: ACVR2B: Activin receptor type-2B; AK1: Adenylate Kinase 1; ANGPTL2: Angiopoietin-like protein 2; AZGP1: Zinc-α-2-glycoprotein; BST2: Bone marrow stromal antigen 2; CA 19-9: Carbohydrate antigen 19-9; CEA: Carcinoembryonic antigen; COL3A1: Collagen alpha-1(III); COL6A3: Collagen alpha-3(VI) chain; CRMP-2: Collapsin response mediator protein-2; DK: Dermokine; GDF15: Growth/differentiation factor 15; GRN: Granulin; GTF2i: General transcription factor; FGFR4: Fibroblast growth factor receptor 4; HMGB1: High mobility group protein B1; IGFBP2: Insulin like growth factor binding protein 2; LAMB1: Laminin β-1; LRG1: Leucine-rich alpha-2-glycoprotein; MAC-2BP/TAA90K: Mac-2 binding protein/Tumor-associated antigen 90 K; MAPKAPK3: Mitogen-activated protein kinase-activated protein kinase 3; MAPRE1: Microtubule-associated protein RP/EB family member 1; MRC1: Macrophage mannose receptor 1; MST1/STK4: Mammalian STE20-like protein kinase 1; NHSL1: NHS-like protein 1; PAI-1: Plasminogen activator inhibitor-1; PIM1: Serine/threonine-protein kinase pim-1; S-p53: Serum-p53; S100A8/A9: S100 calcium-binding protein A8/A9; SEC61β: Protein transport protein Sec61 subunit beta; SERPINI1: Neuroserpin; SREBF2: Sterol regulatory element binding protein 2; SULF1: Sulfatase 1; TFF3: trefoil factor; TIMP1: Tissue inhibitor of metalloproteinases 1; TRFM: Melanotransferrin; -: means not specified by the study; x: this type of protein was identified by the study.

**Table 4:** Diagnostic performance of tumor tissue proteome/TIF markers validated in blood.

First Author, Year, [Ref]	Proteins											Sensitivity (95%CI) %	Specificity (95%CI) %	Area under the Curve (95%CI)	Method	Validation
	CEA	CA19-9	ACVR2B	AZGP1	GDF15	LRG1	S100A9	MAPKAP3	TIMP1	PIM1	Others					
Tumor Tissue Proteome Combination Marker Studies																
Broll, 2001 [56]	x										VEGF	62 (53–71)	85 (74–93)	-	ELISA	No
Alberthsen, 2006 [60]											HNP1, HNP2, HNP3	80 (72–87)	53 (35–70)	-	ELISA	No
Yoneda, 2009 [41]	x	x									Cystatin SN	63 (55–71)	90 (76–97)	-	ELISA	No
Kijanka, 2010 [62]											ZNF700, TSLC1, LASS5, p53, TCF3, SNP29, ZNF638, ICLN, ZNF346, AOPJ75, HMGB1, BAC85857	84 (70–93)	80 (64–91)	-	PrA	SS
Xie, 2010 [27] ^#^											A1AT, CTSD	100 (92–100)	97 (87–100)	-	WB	No
Hamelin, 2011 [61]	x	x									HSP60	47 (38–57)	90 (82–95)	0.77 (0.70–0.84)	ELISA	SS
Kuo, 2011 [39]	x										PLSCR1	85 (73–93)	48 (34–62)	0.80	WB	No
Matsubara, 2011 [42]	x										Adipophilin	31 (15–52)	95 (88–99)	0.783	RPPM	SS
Wang, 2013 [25]	x										Kininogen-1	22 (16–30)	93 (85–97)	-	ELISA	CV
Jiang, 2014 [23]											DC-SIGN, DC-SIGNR	99 (96–100)	95 (89–98)	0.989	ELISA	No
Xue, 2014 [29]	x	x		x								74 (65–82)	73 (57–86)	0.805 (0.738–0.872)	ELISA	LOOCV
Surinova, 2015 [58]						x			x		CP, PON1, SERPINA3	70 (63–76)	79 (67–88)	0.84 (0.75–0.92) *	SRM	TFCV
Wang Y, 2016 [32]						x					TUBB5	63 (36–85)	81 (54–96)	0.74	MRM	No
Xie, 2016 [34]											CELA1, CEL2A, CTRL, TRY2	87 (70-96)	83 (65-94)	0.90 (0.83–0.98)	MRM	No
Tumor Tissue Proteome Individual Marker Studies																
Roessler, 2005 [57]	x										NNMT	51 (41–61) 39 (30–49)	95 (92–97) 95 (92–97)	0.84 0.78	ELISA	No
Kim, 2009 [47]	x						x				S100A8	41 (30–53) 44 (33–56) 22 (13–33)	95 (76–100) 95 (76–100) 100 (94–100)	0.91 0.89 0.78	WB	No
Ji, 2010 [45]	x										ESM-1	99 (95–100) 48 (38–58)	73 (62–82) 99 (94–100)	0.94 0.733	ELISA	No
Han, 2011 [21]											STOML2	71 (59–81)	63 (51–74)	0.77	ELISA	No
Fijneman, 2012 [63]	x										CHI3L1	75 (35–97) 38 (9–76)	89 (74–97) 100 (90–100)	0.81 0.86	ELISA	No
Hosono, 2012 [44] ^A^											TNF-R1	93 (84–98)	82 (65–93)	-	ELISA	No
Yao, 2012 [30]	x										EFEMP2	83 (75–89) 63 (54–72)	93 (85–98) 77 (66–86)	0.923 (0.885–0.961) 0.728 (0.659–0.797)	ELISA	SS
Ji, 2013 [22]				x							PEDF PRDX2	100 (99–100) 34 (29–39) 87 (83–90)	79 (69–87) 96 (89–99) 51 (40–62)	-	ELISA	SS
Niewiarowska, 2014 [64]									x			67 (51–81)	67 (45–85)	0.666	ELISA	No
Sole, 2014 [55] ^§^											COL10A1	63 (52–74)	85 (75–92)	0.75	ELISA	SS

Note: ^#^: The sensitivity and specificity values are for tissue array based validation and not serum based; *: The confidence interval for area under the curve in this study was reported for 99% CI; ^§^: The sensitivity and specificity values are for CRC or Adenoma cases; ^A^: The sensitivity and specificity values are for only Adenoma cases and not CRC; -: means not specified by the study; x: this type of protein was identified by the study. Abbreviation: A: Adenoma; CV: Cross-validation method; ELISA: Enzyme-linked immunosorbent assay; LOOCV: Leave-one-out cross validation; MRM: Multiple reaction monitoring; PrA: Protein Array; RPPM: Reverse phase protein microarray; SRM: Selected reaction monitoring used in tandem mass spectrometry; SS: Split Sampling method; TFCV: Ten-fold Cross validation; WB: Western Blot. Abbreviations for proteins: A1AT: α1 antitrypsin; ACVR2B—Activin receptor type-2B; AOPJ75: KIAA0310 protein; AZGP1: Zinc-α-2-glycoprotein; BAC85857: Unnamed protein product; CA 19-9: Carbohydrate antigen; CEA: Carcinoembryonic antigen 19-9; CELA1: chymotrypsin-like elastase 1; CELA2: chymotrypsin-like elastase 2A; CHI3L1: Chitinase-3-like protein 1; COL10A1: Collagen alpha-1(X) chain; CP: Ceruloplasmin; CTRL: chymopasin; CTSD: Cathepsin D; DC-SIGN: Dendritic cell-specific ICAM-3 grabbing nonintegrin; DC-SIGNR: DC-SIGN-related protein/L-SIGN, CD209L; GDF15: Growth/differentiation factor 15; EFEMP2: EGF-containing fibulin-like extracellular matrix protein 2; ESM-1: Endothelial cell-specific molecule-1; HMGB1: High mobility group protein B1; HNP1: Human neutrophil peptide 1; HNP2: Human neutrophil peptide 2; HNP3: Human neutrophil peptide 3; HSP60: Heat shock protein 60; ICLN: Methylosome subunit plCln; LASS5: Longevity assurance gene homologous 5; LRG1: Leucine-rich alpha-2-glycoprotein; MAPKAPK3: Mitogen-activated protein kinase-activated protein kinase 3; NNMT: Nicotinamide N-Methyltransferase; PEDF: Pigment epithelium derived factor; PIM1: Serine/threonine-protein kinase pim-1; PLSCR1: phospholipid scramblase 1; PON1: Serum paraoxonase/arylesterase 1; PRDX2: Peroxiredoxin 2; S100A8/A9: S100 calcium-binding protein A8/A9; SERPINA3: Serpin peptidase inhibitor clade A; SNP29: Synaptosomal-associated protein 29; STOML2: Stomatin like 2; TCF3: E2A immunoglobulin enhancer-binding factor E12/E47; TIMP1: Tissue inhibitor of metalloproteinases 1; TNF-R1: Tumor necrosis factor receptor 1; TRY2: trypsin 2; TSLC1: Tumour suppressor in lung cancer I; TUBB5: tubulin beta-5 chain; VEGF: Vascular endothelial growth factor; ZNF346: Zinc finger protein 346; ZNF638: Zinc finger protein 638; ZNF700: Zinc finger protein 700.

## Supplementary Materials

The following are available online at http://www.mdpi.com/2072-6694/9/11/156/s1. File S1: Data source and search strategy, Table S2: Stage specific diagnostic performance, Table S3: Function and location of proteins as identified from Uniprot database, Table S4: QUADAS-2 risk of bias assessment of the studies, Table S5: PRISMA 2009 checklist.
