# Supplementary material for: Cell Line Secretome and Tumor Tissue Proteome Markers for Early Detection of Colorectal Cancer: A Systematic Review
Source: Cancers (Basel). 2017 Nov 16;9(11):156. doi: 10.3390/cancers9110156 (PMC5704174; doi:10.3390/cancers9110156)
Supplement: Supplementary file 1 [file cancers-09-00156-s001.pdf]

Supplementary Materials: Cell Line Secretome and Tumor Tissue Proteome Markers for Early Detection of Colorectal Cancer: A Systematic Review

Megha Bhardwaj, Vanessa Erben, Petra Schrotz-King and Hermann Brenner

Supplementary Material File S1: Data sources and search strategy

for PubMed

"Colorectal neoplasm" OR "colon neoplasm" OR "colonic neoplasm" OR "Rectal Neoplasm" OR "colorectal cancer" OR "colon cancer" OR "colonic cancer" OR CRC OR "Colorectal tumor" OR "colon tumor" OR "colonic tumor" OR adenoma
AND
"Cell Line, Tumor"[Mesh] OR "Tumor Microenvironment"[Mesh] OR secretome OR Tissue
AND
"Proteins"[Mesh] OR Proteome OR Proteomics
AND
"Diagnosis" [Mesh] OR sensitivity OR specificity OR AUC OR "area under the curve"
AND
Blood OR Plasma OR Serum
NOT
Therapy OR Treatment OR Progression

As on 18.07.2017-
Pubmed: 1498 records

for Web of Science

TOPIC: (Colorectal neoplasm OR colon neoplasm OR colonic neoplasm OR Rectal Neoplasm OR colorectal cancer OR colon cancer OR colonic cancer OR CRC OR Colorectal tumor OR colon tumor OR colonic tumor OR adenoma)
AND
TOPIC: (Cell Line OR Tumor OR Tumor Microenvironment OR secretome OR Tissue)
AND
TOPIC: (Proteins OR Proteome OR Proteomics)
AND
TOPIC: (Diagnosis OR sensitivity OR specificity OR AUC OR area under the curve)
AND
TOPIC: (Blood OR Plasma OR Serum)
NOT
TOPIC: (Therapy OR Treatment OR Progression)

As on 18.07.2017-
Web of Science: 865 records

Table S2. Stage specific diagnostic performance

First author, Year [Ref]	Number (N)							Sensitivity						Specificity					
	Ad	AA	0	I/A	II/B	III/C	IV/D	A	AA	I/A	II/B	III/C	IV/D	A	AA	I/A	II/B	III/C	IV/D
			Early			Late				Early		Late				Early		Late	
Broll, 2001 [56]				41	27	30	24												
Nam, 2005 [66]																			
Alberthsen, 2006 [60]				15	46	38	20					78						77	
Roessler, 2005 [57]			3	33	23	21	23												
Wu, 2008 [35]				27	72	60	40												
Wu, 2008 [36]				23	90	86	81												
Babel, 2009 [50]				6	14	20	12												
Kim, 2009 [47]<br>S100A8<br>S100A9<br>CEA	11			14	23	21	19			32<br>40<br>21						95<br>95<br>100			
Yoneda, 2009 [41]				40	51	40	26			43	61	68	92						
Ji, 2010 [45]	10			14	37	39	10												
Kijanka, 2010 [62]																			
Tagi, 2010 [40]			6	28	25	27	19			58	44	63	82			80	80	80	80
Xie, 2010 [27]<br>TFF3<br>GDF15													74<br>83						91<br>82
Xue, 2010 [28]				68		76													
Babel, 2011 [51]				6	11	16	17												
Fan, 2011 [38]				10	25	35	16			71		71							
Hamelin, 2011 [61]				27	29	29	27												
Han, 2011 [21]																			
Kuo, 2011 [39]				25		34				80		88							
Matsubara, 2011 [42]				12	5	8	1												
Weiss, 2011 [59]					15	18	26												
Barderas, 2012 [52]				6	11	16	17			88						83			
Fijneman, 2012 [63]<br>CHI3L1<br>CEA	20	22			1	4	1	25<br>5	55<br>5					89<br>10<br>0	89<br>100				
Hosono, 2012 [44]	62							93						82					
Ladd, 2012 [65]				4	13	12	3												
Lee, 2012 [48]				34	83	70	32				47					87			
Rodriguez-P, 2012 [54]				6	13	5	7												
Yao, 2012]30]	14																		

Barderas, 2013[53]				6	7	12	15												
Ji, 2013 [22]	45			102	110	109	84												
Wang, 2013 [25]<br>Kininogen-1<br>CEA				14	63	37	29			70<br>39		73<br>35				66<br>86		66<br>86	
Jiang, 2014 [23]<br>DC-SIGN<br>DC-SIGNR<br>CEA<br>CA19-9				5	68	44	69			81<br>49<br>19<br>10						56<br>93<br>92<br>95			
Niewiarowska, 2014 [64]				1	4	38													
Shin, 2014 [46]	11			68	68	65	27												
Sole, 2014 [55]	23			9	27	34	10												
Toiyama, 2014 [43]				48	55	54	38				54					93			
Xue, 2014 [29]				20	56	26	18												
Chiang, 2015 [37]				61		59													
Lin, 2015 [49]				6	9	22	8												
Qiao, 2015 [24]																			
Surinova, 2015 [58]				43	58	49	52												
Taguchi, 2015 [67]	60			11	19	21	9	35		47		50		95		95		95	
Zhang, 2015 [33]																			
Fan, 2016 [31]																			
Wang X, 2016 [26]																			
Wang Y, 2016 [32]					7	9													
Xie, 2016 [34]																			

Abbreviations: Ad- Adenoma, AA- Advanced adenoma, ACA- Adenocarcinoma. Note- Stages I/II/III/IV as per UICC classification of tumors and stages A/B/C/D as per AJCC classification.

Table S3. Function and location of proteins as identified from UniProt database [19].

Protein	Molecular Function	Biological Function	Location	Cellular component
**A1AT**	serine protease inhibitor, glycoprotein binding	Acute phase, Blood coagulation, Hemostasis	secreted	endoplasmic reticulum, extracellular matrix
**ACVR2B**	ATP binding, growth factor binding, protein serine/threonine/tyrosine kinase activity	activation of protein kinase activity	cell membrane	cytoplasm
**Adipophilin**	development and maintenance of adipose tissue	lipid storage and long-chain fatty acid transport	membrane	endoplasmic reticulum, cytosol
**AK1**	Kinase, Transferase	ATP-binding, Nucleotide-binding	cytoplasm	extracellular exosome
**ANGPTL2**	receptor binding	multicellular organism development	secreted	extracellular space
**AOPJ75**				
**AZGP1**	antigen binding, glycoprotein binding	antigen processing and presentation, cell adhesion	secreted	extracellular exosome
**α-defensin 6**	Antibiotic, Antimicrobial, Defensin, Fungicide	antimicrobial humoral immune response	secreted	extracellular region
**BAC85857**				
**BST2**	metalloendopeptidase inhibitor activity, protein homodimerization activity, RNA binding	Antiviral defense, B-cell activation, Immunity, Innate immunity	golgi apparatus	extracellular exosome
**CA 19-9**	glycoprotein	tumor marker		
**CEA**	glycoprotein	tumor marker		
**CELA1**	Hydrolase, Protease, Serine protease, metal-ion binding	inflammatory response, elastin catabolic process	secreted	extracellular space
**CELA2**	Hydrolase, Protease, Serine protease, metal-ion binding	cornification	secreted	extracellular space
**CHI3L1**	Antimicrobial, carbohydrate binding, extracellular matrix structural constituent	Apoptosis, Inflammatory response	secreted	cytoplasm, extracellular space
**Clusterin**	Chaperone	Apoptosis, Complement pathway, Immunity, Innate immunity	secreted	cytoplasm, extracellular matrix
**COL10A1**	metal ion binding	cartilage development, collagen catabolic process	secreted	extracellular matrix
**COL3A1**	calcium binding, metal ion binding, integrin binding	aging, cell-matrix adhesion, collagen catabolic process	secreted	extracellular matrix
**COL6A3**	Protease inhibitor, Serine protease inhibitor	Cell adhesion	secreted	extracellular matrix
**CP**	chaperone binding, copper ion binding, oxidoreductase	Copper transport, Ion transport, Transport	secreted	extracellular exosome
**CRMP2**	Developmental protein	Differentiation, Neurogenesis	cytoplasm	cytoskeleton, extracellular exosome
**CTRL**	Hydrolase, Protease, Serine protease	digestion, proteolysis	secreted	extracellular space
**CTSD**	Aspartyl protease, Hydrolase, Protease		lysosome, melanosome	extracellular exosome
**Cystatin SN**	Thiol protease inhibitor	detection of chemical stimulus involved in sensory perception of bitter taste	secreted	extracellular space
**DC-SIGN**	Host cell receptor for virus entry, Receptor	Adaptive immunity, Cell adhesion, Endocytosis, Host-virus interaction, Immunity, Innate immunity	cytoplasm	extracellular exosome
**DC-SIGNR**	Host cell receptor for virus entry, Receptor	Adaptive immunity, Cell adhesion, Endocytosis, Host-virus interaction, Immunity, Innate immunity	cytoplasm	extracellular exosome
**DK**	soluble regulator of keratinocyte differentiation	cornified envelope assembly	secreted	extracellular exosome
**Protein**	**Molecular Function**	**Biological Function**	**Location**	**Cellular component**
**EFEMP2**	calcium ion binding, extracellular matrix structural constituent		secreted	extracellular exosome

**ESM-1**	hepatocyte growth factor receptor binding	Angiogenesis	secreted	extracellular region
**FGFR4**	Kinase, Receptor, Transferase, Tyrosine-protein kinase	ATP-binding, Nucleotide-binding	secreted	endoplasmic reticulum, cytoplasm
**GDF15**	Cytokine activity, Growth factor/transforming growth factor beta receptor binding	BMP signaling pathway, cell-cell signaling, cell development	secreted	extracellular exosome
**GRN**	Cytokine activity, Growth factor activity		secreted	endoplasmic reticulum, extracellular exosome
**GTF2i**	DNA-binding	Transcription, Transcription regulation	cytoplasm	nucleus, cytoplasm
**HMGB1**	DNA-binding	Adaptive immunity, Autophagy, Chemotaxis, DNA damage, DNA recombination, DNA repair, Immunity, Inflammatory response, Innate immunity	cytoplasm	cell surface, extracellular exosome
**HNP1**	Antibiotic, Antimicrobial, Defensin, Fungicide	Antiviral defense	secreted	extracellular exosome
**HNP2**	Antibiotic, Antimicrobial, Defensin, Fungicide	Antiviral defense	secreted	extracellular exosome
**HNP3**	Antibiotic, Antimicrobial, Defensin, Fungicide	Antiviral defense	secreted	extracellular exosome
**HSP60**	Chaperone, Hydrolase, ATPbinding	Host-virus interaction	mitochondrial matrix	cytoplasm, mitochondrion
**ICLN**	RNA-binding	cell volume homeostasis	cytoplasm	nucleus, cytoplasm
**IGFBP2**	Growth factor binding	Growth regulation	secreted	extracellular exosome
**Kininogen-1**	Protease inhibitor, Thiol protease inhibitor,, Vasodilator	Blood coagulation, Hemostasis, Inflammatory response	secreted	extracellular exosome
**LAMB1**	extracellular matrix structural constituent	cell adhesion	secreted	extracellular matrix
**LASS5**	DNA-binding, Transferase	Lipid biosynthesis, Lipid metabolism, Sphingolipid metabolism	endoplasmic reticulum	nucleus
**LRG1**	transforming growth factor beta receptor binding	brown fat cell differentiation, neutrophil degranulation	secreted	extracellular exosome
**Mac-2BP**	5cavenger receptor activity	cell adhesion	secreted	extracellular matrix
**MAPKAPK3**	Kinase, Serine/threonine-protein kinase, Transferase	ATP-binding, Nucleotide-binding	nucleus, cytoplasm	nucleus, cytoplasm
**MAPRE1**	cadherin binding Source, identical protein binding	Cell cycle, Cell division, Mitosis	cytoplasm	Cytoplasm, Microtubule
**MRC1**	Host cell receptor for virus entry, Receptor	Endocytosis, Host-virus interaction	cell membrane	endosome
**MST1/STK4**	Kinase, Serine/threonine-protein kinase, Transferase	Apoptosis	cytoplasm	nucleus, cytoplasm
**NHSL1**		motor neuron migration		membrane
**NNMT**	Methyltransferase, Transferase	methylation	cytoplasm	cytoplasm
**p53**	Activator, DNA-binding, Repressor	Apoptosis, Biological rhythms, Cell cycle, Host-virus interaction, Necrosis, Transcription, Transcription regulation	cytoplasm	nucleus, cytoplasm
**PAI-1**	Protease inhibitor, Serine protease inhibitor	angiogenesis, cellular response to lipopolysaccharide	secreted	extracellular exosome
**PEDF**	serine-type endopeptidase inhibitor activity	aging, cell proliferation	secreted	melanosome, extracellular exosome
**Protein**	**Molecular Function**	**Biological Function**	**Location**	**Cellular component**
**PIM1**	Kinase, Serine/threonine-protein kinase, Transferase	Apoptosis, Cell cycle	cytoplasm	cell membrane
**PLSCR1**	DNA-binding	Antiviral defense	cell membrane	extracellular exosome
**PON1**	Hydrolase	aromatic compound catabolic process, carboxylic acid catabolic process, cholesterol metabolic process	secreted	extracellular space

**PRDX2**	Antioxidant, Oxidoreductase, Peroxidase	cellular response to oxidative stress	cytoplasm	cytoplasm
**S100A8**	Antimicrobial	Apoptosis, Autophagy, Chemotaxis, Immunity, Inflammatory response, Innate immunity	secreted	cytoplasm, extracellular space
**S100A9**	Antimicrobial, Antioxidant	Apoptosis, Autophagy, Chemotaxis, Immunity, Inflammatory response, Innate immunity	secreted	cytoplasm, extracellular space
**SEC61β**	epidermal growth factor binding	Protein transport, Translocation, Transport	cytosol	endoplasmic reticulum
**sE-cadherin**	Cell adhesion molecule binding	Cell adhesion	cell membrane	golgi apparatus
**SERPINA3**	Protease inhibitor, Serine protease inhibitor	acute-phase response, inflammatory response	secreted	extracellular exosome
**SERPINI1**	Protease inhibitor, Serine protease inhibitor	central & peripheral nervous system development	secreted	extracellular exosome
**SNP29**		Autophagy, Cilium biogenesis/degradation, Protein transport, Transport	cytoplasm	cell membrane
**Spondin-2**	Antigen binding	Cell adhesion	secreted	extracellular space
**SREBF2**	Activator, DNA-binding	Cholesterol metabolism, Lipid metabolism, Steroid metabolism, Sterol metabolism, Transcription, Transcription regulation	cytoplasm	endoplasmic reticulum, cytoplasm
**STOML2**	cardiolipin binding, GTPase binding, receptor binding		cell membrane	cell membrane, cytoskeleton
**SULF1**	Hydrolase	Apoptosis	endoplasmic reticulum	cell surface
**TCF3**	DNA-binding	Differentiation, Neurogenesis, Transcription regulation	nucleus	cytoplasm
**TFF3**		defense response, wound healing	secreted	cytoplasm, extracellular matrix
**TIMP1**	Growth factor, Metalloenzyme inhibitor, Metalloprotease inhibitor, Protease inhibitor	aging, cartilage development, cell activation	secreted	extracellular exosome
**TNF-R1**	Receptor	Apoptosis, Host-virus interaction	secreted	golgi apparatus
**TRFM**	iron ion binding	Ion transport, Iron transport, Transport	cell membrane	cell membrane
**TRY2**	hydrolase, protease, Serine protease, calcium-ion binding	anti-microbial humoral response, digestion	secreted	extracellular space
**TSLC1**	Developmental protein	Apoptosis, Cell adhesion, Differentiation, Immunity, Spermatogenesis	extracellular exosome	cell membrane
**TUBB5**	GTPase activity and binding	cell division, cell process	cytoplasm	cytoplasm
**VEGF**	Developmental protein, Growth factor, Heparin-binding,	Angiogenesis, Differentiation	secreted	extracellular space
**ZNF346**	RNA-binding	positive regulation of apoptotic process	nucleus	cytoplasm, nucleus
**ZNF638**	Activator, DNA-binding, RNA-binding	Transcription, Transcription regulation	nucleus speckle	nucleus, cytoplasm
**ZNF700**	DNA-binding	Transcription, Transcription regulation	nucleus	nucleus, cytoplasm

Table S4. QUADAS-2 risk of bias assessment of the studies [18].

Study	Risk of Bias				Applicability Concerns		
	Patient Selection	Index Test	Reference StandarD	Flow and Timing	Patient Selection	Index Test	Reference Standard
Broll, 2001 [56]	☹	?	☹	?	?	☺	?
Nam, 2005 [66]	☹	?	?	?	☺	☺	?
Albrethsen, 2006 [60]	☹	☹	?	?	☺	☺	?
Roeßler, 2005 [57]	☹	☹	☹	☹	☹	☺	?
Wu, 2008 [35]	☹	☹	☹	?	?	☺	?
Wu, 2008 [36]	☹	☹	☹	?	?	☺	?
Babel, 2009 [50]	☹	?	☹	?	?	☺	?
Kim, 2009 [47]	☹	☹	☹	?	?	☺	?
Yoneda, 2009 [41]	☹	☹	☺	☹	?	☺	☺
Ji, 2010 [45]	☹	☹	☹	?	☺	☺	?
Kijanka, 2010 [62]	☹	☺	☺	☺	☺	☺	☺
Tagi, 2010 [40]	☹	?	?	?	☹	☺	?
Xie, 2010 [27]	☹	?	☹	?	☺	☺	?
Xue, 2010 [28]	☹	☹	☹	?	☺	☺	?
Babel, 2011 [51]	☹	☺	☹	?	?	☺	?
Fan, 2011 [38]	☹	?	☹	?	☺	☺	?
Hamelin, 2011 [61]	☹	?	☹	?	?	☺	?
Han, 2011 [21]	☹	☹	☹	?	☺	☺	?
Kuo, 2011 [39]	☹	?	☹	?	?	☺	?
Matsubara, 2011 [42]	☹	?	☹	?	☹	☺	?
Weiss, 2011 [59]	☹	?	☹	?	?	☺	?
Barderas, 2012 [52]	☹	☺	?	?	?	☺	?
Fijneman, 2012 [63]	☹	☹	☺	☺	☺	☺	☺
Hosono, 2012 [44]	☹	☹	☺	☺	☺	☺	☺
Ladd, 2012 [65]	☺	☹	?	?	☺	☺	?
Lee, 2012 [48]	☹	☹	☺	☺	☺	☺	☺
Rodriguez-P, 2012 [54]	☹	☹	?	?	☺	☺	?
Yao, 2012 [30]	☹	☹	☺	?	?	☺	?
Barderas, 2013 [53]	☹	☹	?	?	?	☺	?
Ji, 2013 [22]	☹	☺	☹	?	☹	☺	?
Wang, 2013 [25]	☹	?	☹	?	?	☺	?
Jiang, 2014 [23]	☹	☹	☹	☹	☹	☺	?
Niewiarowska, 2014 [64]	☹	☹	☹	?	☺	☺	?
Shin, 2014 [46]	☹	?	☹	☹	?	☺	?
Sole, 2014 [55]	☹	☺	☺	☹	☺	☺	☺
Toiyama, 2014 [43]	☹	?	?	?	☺	☺	?
Xue, 2014 [29]	☹	?	?	?	☺	☺	?

Chiang, 2015 [37]	☹	?	☹	☺	☺	☺	?
Lin, 2015 [49]	☹	☹	?	?	?	☺	?
Qiao, 2015 [24]	☹	☹	☹	?	?	☺	?
Surinova, 2015 [58]	☹	☺	☺	☺	☺	☺	☺
Taguchi, 2015 [67]	☹	☺	?	?	☺	☺	?
Zhang, 2015 [33]	☹	☹	☹	?	☺	☺	?
Fan, 2016 [31]	☹	☹	☹	?	☺	☺	?
Wang X, 2016 [26]	☹	☹	☹	☹	?	☺	?
Wang Y, 2016 [32]	☹	☹	?	?	?	☺	?
Xie, 2016 [34]	☹	☹	?	?	?	☺	?

Note: In the above table ☺ represents low risk, ☹ represents high risk and unclear risk is represented as ?

Table S5. PRISMA 2009 Checklist [20].

Section/topic	#	Checklist item	Reported on page #
**Title**			
Title	1	Identify the report as a systematic review, meta-analysis, or both.	1
**Abstract**			
Structured summary	2	Provide a structured summary including, as applicable: background; objectives; data sources; study eligibility criteria, participants, and interventions; study appraisal and synthesis methods; results; limitations; conclusions and implications of key findings; systematic review registration number.	1
**Introduction**			
Rationale	3	Describe the rationale for the review in the context of what is already known.	2
Objectives	4	Provide an explicit statement of questions being addressed with reference to participants, interventions, comparisons, outcomes, and study design (PICOS).	2
**Methods**			
Protocol and registration	5	Indicate if a review protocol exists, if and where it can be accessed (e.g., Web address), and, if available, provide registration information including registration number.	2
Eligibility criteria	6	Specify study characteristics (e.g., PICOS, length of follow-up) and report characteristics (e.g., years considered, language, publication status) used as criteria for eligibility, giving rationale.	2, 5
Information sources	7	Describe all information sources (e.g., databases with dates of coverage, contact with study authors to identify additional studies) in the search and date last searched.	2, S1
Search	8	Present full electronic search strategy for at least one database, including any limits used, such that it could be repeated.	S1
Study selection	9	State the process for selecting studies (i.e., screening, eligibility, included in systematic review, and, if applicable, included in the meta-analysis).	2, 5
Data collection process	10	Describe method of data extraction from reports (e.g., piloted forms, independently, in duplicate) and any processes for obtaining and confirming data from investigators.	2
Data items	11	List and define all variables for which data were sought (e.g., PICOS, funding sources) and any assumptions and simplifications made.	2
Risk of bias in individual studies	12	Describe methods used for assessing risk of bias of individual studies (including specification of whether this was done at the study or outcome level), and how this information is to be used in any data synthesis.	2
Summary measures	13	State the principal summary measures (e.g., risk ratio, difference in means).	2, 7–9
Synthesis of results	14	Describe the methods of handling data and combining results of studies, if done, including measures of consistency (e.g., I^2) for each meta-analysis.	-

Section/topic	#	Checklist item	Reported on page #
Risk of bias across studies	15	Specify any assessment of risk of bias that may affect the cumulative evidence (e.g., publication bias, selective reporting within studies).	7, 27
Additional analyses	16	Describe methods of additional analyses (e.g., sensitivity or subgroup analyses, meta-regression), if done, indicating which were pre-specified.	
**Results**			
Study selection	17	Give numbers of studies screened, assessed for eligibility, and included in the review, with reasons for exclusions at each stage, ideally with a flow diagram.	3, 5
Study characteristics	18	For each study, present characteristics for which data were extracted (e.g., study size, PICOS, follow-up period) and provide the citations.	3, 11–12
Risk of bias within studies	19	Present data on risk of bias of each study and, if available, any outcome level assessment (see item 12).	4-10
Results of individual studies	20	For all outcomes considered (benefits or harms), present, for each study: (a) simple summary data for each intervention group (b) effect estimates and confidence intervals, ideally with a forest plot.	14–15
Synthesis of results	21	Present results of each meta-analysis done, including confidence intervals and measures of consistency.	-
Risk of bias across studies	22	Present results of any assessment of risk of bias across studies (see Item 15).	SF4
Additional analysis	23	Give results of additional analyses, if done (e.g., sensitivity or subgroup analyses, meta-regression [see Item 16]).	-
**Discussion**			
Summary of evidence	24	Summarize the main findings including the strength of evidence for each main outcome; consider their relevance to key groups (e.g., healthcare providers, users, and policy makers).	14–15
Limitations	25	Discuss limitations at study and outcome level (e.g., risk of bias), and at review-level (e.g., incomplete retrieval of identified research, reporting bias).	15
Conclusions	26	Provide a general interpretation of the results in the context of other evidence, and implications for future research.	15
**Funding**			
Funding	27	Describe sources of funding for the systematic review and other support (e.g., supply of data); role of funders for the systematic review.	15
